# Variation in Fumonisin and Ochratoxin Production Associated with Differences in Biosynthetic Gene Content in *Aspergillus niger* and *A. welwitschiae* Isolates from Multiple Crop and Geographic Origins

**DOI:** 10.3389/fmicb.2016.01412

**Published:** 2016-09-09

**Authors:** Antonia Susca, Robert H. Proctor, Massimiliano Morelli, Miriam Haidukowski, Antonia Gallo, Antonio F. Logrieco, Antonio Moretti

**Affiliations:** ^1^Institute of Sciences of Food Production, National Research Council of ItalyBari, Italy; ^2^United States Department of Agriculture-Agricultural Research Service National Center for Agricultural Utilization ResearchPeoria, IL, USA; ^3^Institute for Sustainable Plant Protection, Institute for Sustainable Plant Protection, UOS BariBari, Italy; ^4^Institute of Sciences of Food Production, National Research Council of ItalyLecce, Italy

**Keywords:** *fum* cluster, *ota* cluster, fumonisin, ochratoxin, biosynthetic gene cluster, *Aspergillus niger*, *Aspergillus welwitschiae*

## Abstract

The fungi *Aspergillus niger* and *A. welwitschiae* are morphologically indistinguishable species used for industrial fermentation and for food and beverage production. The fungi also occur widely on food crops. Concerns about their safety have arisen with the discovery that some isolates of both species produce fumonisin (FB) and ochratoxin A (OTA) mycotoxins. Here, we examined FB and OTA production as well as the presence of genes responsible for synthesis of the mycotoxins in a collection of 92 *A. niger*/*A. welwitschiae* isolates from multiple crop and geographic origins. The results indicate that (i) isolates of both species differed in ability to produce the mycotoxins; (ii) FB-nonproducing isolates of *A. niger* had an intact fumonisin biosynthetic gene (*fum*) cluster; (iii) FB-nonproducing isolates of *A. welwitschiae* exhibited multiple patterns of *fum* gene deletion; and (iv) OTA-nonproducing isolates of both species lacked the ochratoxin A biosynthetic gene (*ota*) cluster. Analysis of genome sequence data revealed a single pattern of *ota* gene deletion in the two species. Phylogenetic analysis suggest that the simplest explanation for this is that *ota* cluster deletion occurred in a common ancestor of *A. niger* and *A. welwitschiae*, and subsequently both the intact and deleted cluster were retained as alternate alleles during divergence of the ancestor into descendent species. Finally, comparison of results from this and previous studies indicate that a majority of *A. niger* isolates and a minority of *A. welwitschiae* isolates can produce FBs, whereas, a minority of isolates of both species produce OTA. The comparison also suggested that the relative abundance of each species and frequency of FB/OTA-producing isolates can vary with crop and/or geographic origin.

## Introduction

*Aspergillus niger* is one of the most important filamentous fungi used for biotechnological purposes and has been labeled by the US Food and Drug Administration as “generally recognized as safe” (GRAS). It is widely used in industrial fermentation of organic acids and enzymes, particularly citric acid and extracellular enzymes, and in fermentation of some oriental foods and beverages. The fungus is also a common member of microbial communities on food crops (Varga et al., 2011; Susca et al., 2013, 2014a,b). *A. niger* is part of a taxonomic grouping of at least 11 species known as the *A. niger* “aggregate,” which in turn is part of formal taxon *Aspergillus* section *Nigri*, known less formally as the black aspergilli (Varga et al., 2011). Species of black aspergilli are morphologically similar, and in some cases indistinguishable, but can be reliably distinguished by DNA sequence analysis of housekeeping genes, such as the calmodulin and beta tubulin genes (Perrone et al., 2011; Hong et al., 2013). Among species of the black aspergilli, only *A. niger* and its sister species *A. welwitschiae* have been reported to produce both fumonisin (FB) (Frisvad et al., 2007, 2011; Mogensen et al., 2010) and ochratoxin A (OTA) (Medina et al., 2005; Battilani et al., 2006). Due to the toxicity of these mycotoxins, there is a need to assess the potential risk that FB and OTA-producing black aspergilli pose to human health. Such assessments will be aided by information on production of the mycotoxins by *Aspergillus* strains used in food and beverage fermentation, mycotoxin production during fermentation processes, frequencies of producing and nonproducing isolates in microbial communities on crops, and the genetic bases for production vs. nonproduction.

**Figure 1:**
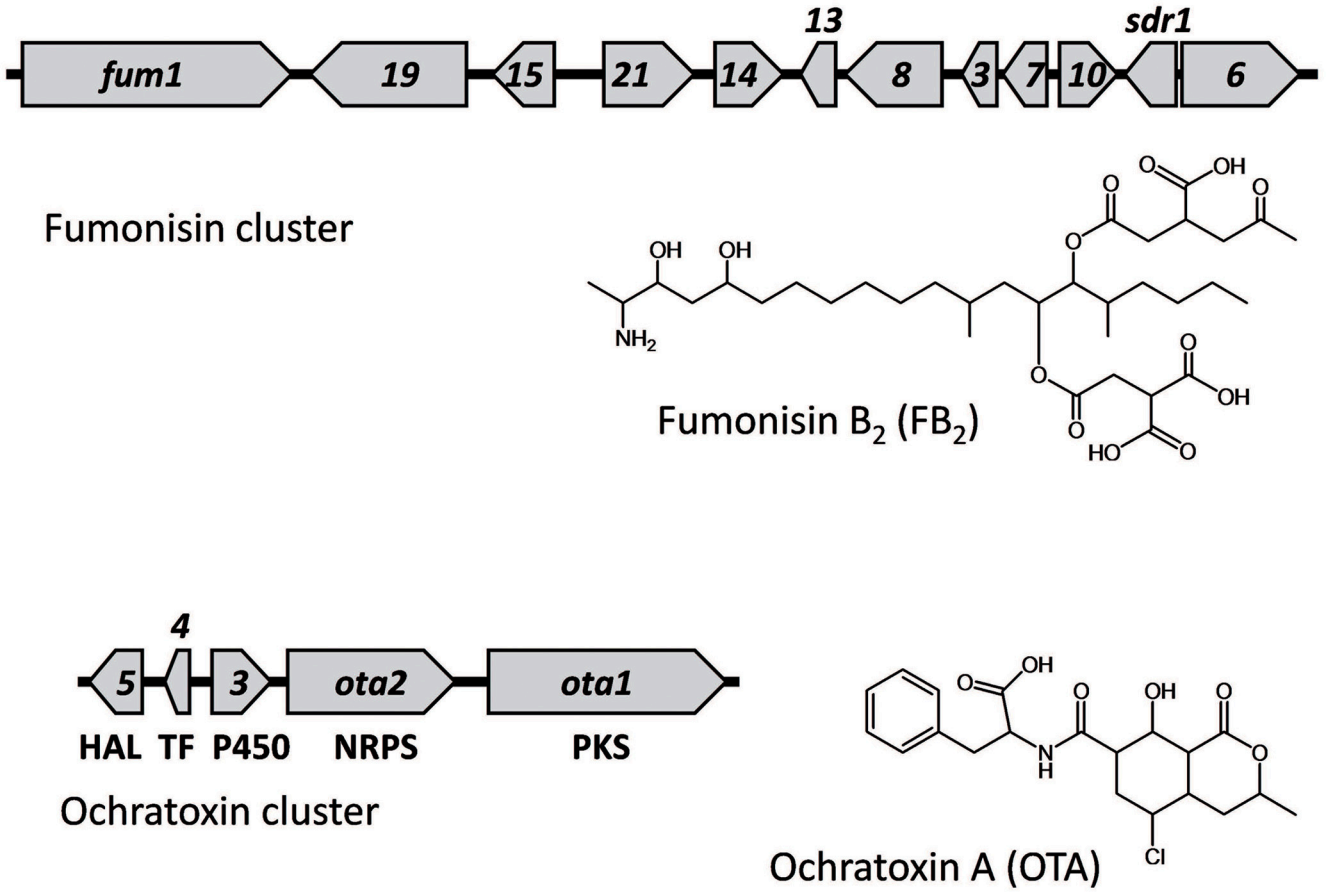
**The fumonisin biosynthetic (***fum***) and ochratoxin biosynthetic (***ota***) gene clusters in ***A. niger***, as well as structures of an analog of each of the corresponding mycotoxins**. Arrows represent genes and point in the direction of transcription. Due to space limitations, only numbers are given for some *fum* and *ota* genes (i.e., in the *fum* cluster, *19, 15*, and *21* indicate *fum19, fum15*, and *fum21* respectively). The predicted functions of the *ota* genes based on sequence homology to genes of known function are indicated below each gene: PKS for polyketide synthase; NRPS for non-ribosomal peptide synthetase; P450 for cytochrome P450 monooxygenase; TF for bZIP transcription factor, and HAL for halogenase. *ota1, ota2, ota3, ota4*, and *ota5* correspond to gene models An15g07880, An15g07890, An15g07900, An15g07910, and An15g07920, respectively, in *A. niger* strain CBS 513.88 (Pel et al., 2007; Ferracin et al., 2012) and gene models 151162, 402289, 398584, 407763, and 51750, respectively, in the genome database for *A. carbonarius* strain ITEM 5010 at the Joint Genome Institute (http://jgi.doe.gov/carbonarius/) (Nordberg et al., 2014). In the *fum* cluster, the gene labeled as *s* is the dehydrogenase gene *sdr1* (Susca et al., 2014a).

The genome sequence of *A. niger* revealed the presence of homologs of the fumonisin (*fum*) and ochratoxin (*ota*) biosynthetic gene clusters that had been described in other fungi (Geisen et al., 2006; Alexander et al., 2009; Gallo et al., 2009). The *A. niger fum* cluster consists of homologs of 10 genes that had been previously characterized in the *Fusarium fum* cluster and an additional gene, *sdr1*, that is not present in *Fusarium* (Figure 1; Baker, 2006; Susca et al., 2014a). The functions of *ota* genes in ochratoxin biosynthesis have not been characterized as extensively as *fum* genes in fumonisin biosynthesis. Only the polyketide synthase gene (for convenience in this study we will refer to it as *ota1*), and the non-ribosomal peptide synthetase (NRPS) gene (*ota2*) have been subjected to functional analysis and demonstrated to be required for ochratoxin production (Geisen et al., 2006; Gallo et al., 2012, 2014). The three other genes, *ota3*–*ota5*, located upstream of *ota1* and *ota2* are hypothesized to be part of the cluster because of their proximity to *ota1* and *ota2*, and because they exhibit expression patterns similar to *ota1* and *ota2* (Ferracin et al., 2012; Gil-Serna et al., 2015). In addition, the presence of a chlorine atom in ochratoxin A is consistent with the predicted halogenase function of the *ota5*-encoded protein (Geisen et al., 2006; Gallo et al., 2012). Likewise, the presence of oxygen atoms in the structures of multiple ochratoxins is consistent with the predicted cytochrome P450 monooxygenase function of the *ota3*-encoded protein. Fungal gene clusters responsible for synthesis of secondary metabolites, including mycotoxins, often include a transcription factor that controls expression of the cluster genes (Keller et al., 2005; Brakhage, 2013). Thus, the predicted bZIP transcription factor function of the *ota4*-encoded protein is also consistent with it being part of the ochratoxin cluster.

Although fumonisin and ochratoxin production have been reported in both *A. niger* and *A. welwitschiae*, not all strains of these species produce the mycotoxins in laboratory culture (Susca et al., 2010, 2014a; Varga et al., 2010; Frisvad et al., 2011; Storari et al., 2012; Palumbo et al., 2013; Gherbawy et al., 2015; Massi et al., 2016). PCR, Southern and genome sequence data indicate that FB-nonproducing strains of *A. welwitschiae* have a partially deleted *fum* cluster. An analysis of a collection of FB-nonproducing isolates of *A. welwitschiae* from grapes grown in the Mediterranean Basin indicated that all the isolates exhibited the same eight-gene deletion within the *fum* cluster (Susca et al., 2014a), whereas, *A. welwitschiae* isolates from raisins produced in California exhibited two different, although similar, patterns of gene deletion within the *fum* cluster (Palumbo et al., 2013). In contrast, the same types of analyses indicated that FB-nonproducing isolates of *A. niger* from Mediterranean grapes and Californian raisins had an intact *fum* cluster (Palumbo et al., 2013; Susca et al., 2014a). Sequence analysis indicated that in the clusters of FB-nonproducing isolates of *A. niger*, the *fum* gene homologs were functional; i.e., the genes did not have insertions, deletions, transitions or transversions predicted to render the genes nonfunctional (Susca et al., 2014a). Together, these findings suggested that the genetic basis for FB nonproduction differs in *A. niger* and *A. welwitschiae*.

Differences in homologs of the *ota* cluster in *A. niger* and *A. welwitschiae* have received less attention than the *fum* cluster. However, the genome sequence of OTA-producing *A. niger* strain CBS 513.88 has an intact *ota* cluster, whereas the genome sequence of OTA-nonproducing strain ATCC 1015 has a 22-kb deletion that includes *ota1*–*ota5* (Andersen et al., 2011; Ferracin et al., 2012). Furthermore, in a recent study of a collection of 89 *A. niger* isolates and 86 *A. welwitschiae* isolates recovered from multiple crops in Brazil, analysis of the presence of *ota1* and *ota5* by PCR indicated that most OTA-nonproducing isolates of both species lacked the two genes.

The objective of the current study was to gain further insight into the biodiversity of *A. niger* and *A. welwitschiae* with respect to variation in OTA and FB production as well as variation in the gene content of the *fum* and *ota* clusters. To meet this objective, we examined a collection of 92 isolates from multiple crops covering a wider range of geographic locations than previously included in one study. The isolates were examined for FB and OTA production as well as for the presence and absence of 10 genes within the *fum* cluster and 4 genes within the *ota* cluster. The findings indicate that mycotoxin production varies among the isolates, provide evidence for novel patterns of gene deletion within the *fum* cluster in FB-nonproducing isolates of *A. welwitschiae*, and provide evidence for deletion of the entire *ota* cluster in OTA-nonproducing isolates of both *A. niger* and *A. welwitschiae*.

## Materials and methods

### Fungal strains

In this study, we examined a collection of 92 fungal isolates that were previously identified by colony morphology as *A. niger-A. welwitschiae*. The isolates were selected to represent strains recovered from a diversity of crop species and geographic origins (**Table 2**). Thirty-four isolates originated from raisins, 32 from grapes, 8 from cashew nuts, 7 from maize, 5 from pistachio, 3 from almonds, and 3 from walnuts. Furthermore, 33 of the isolates were from Turkey, 19 from Italy, 10 from USA, 7 from Brazil, 8 from Argentina, 6 from Greece, 4 from Portugal, 2 from Chile, and one isolate each from China, India, Iran, and Spain. For comparison purposes, the study also included the *A. niger* type strain (ITEM 4501) as well as a second *A. niger* reference strain (ITEM 9568, syn CBS513.88) and a *A. welwitschiae* reference strain (ITEM 4552) (Perrone et al., 2011; Hong et al., 2013). Thirty-six of the isolates from grape were previously identified to species, as well as previously analyzed for fumonisin production and the presence of *fum* genes (Susca et al., 2014a). All isolates were obtained from the Agri-Food Toxigenic Fungi Culture (ITEM) collection at the Institute of Sciences of Food Production CNR-ISPA. Detailed information about the strain features (year of isolation, depositor, toxin production capability, etc.) are available from the ITEM database (http://www.ispa.cnr.it/Collection).

### Growth conditions and DNA extraction

For each strain, conidia were scraped from the surface of PDA cultures and inoculated in 100 ml of Wikerham's medium (40 g of glucose, 5 g of peptone, 3 g of yeast extract, and 3 g of malt extract, distilled water up to 1 L). The resulting liquid cultures were then incubated with shaking (150 rpm) at 25°C for 2 days. Mycelia were filtered, lyophilized and ground using iron beads in a Mill MM 301 mixer (Retsch, Germany). DNA isolation was done with “Wizard® Magnetic Purification System for Food” kit (Promega, USA) according to manufacturer's instructions and starting from 10 mg of ground dried mycelium. Quality and yield of resulting DNA were evaluated by agarose gel electrophoresis, and final quantification was performed with NanoDrop ND-1000 spectrophotometer (Thermo Fisher Scientific, USA).

### Fungal species identification

Species identification was performed through a DNA sequence-based approach by comparing a 650-nt, PCR-amplified fragment of the calmodulin gene (*CaM*) from each isolate against the corresponding sequence from reference strains of *A. niger* (ITEM 4501, AY585536) and *A. welwitschiae* (ITEM 4509, syn CBS 557.65, AJ964874) (Perrone et al., 2011; Hong et al., 2013). PCR products obtained using CL1/CL2A primers (O'Donnell et al., 2000) were purified with the enzymatic mixture EXO/FastAP (Exonuclease I and Thermosensitive Alkaline Phosphatase, Thermo Fisher Scientific, USA) and submitted to bidirectional sequencing with the BigDye® Terminator v3.1 Cycle Sequencing Kit (Applied Biosystems Inc., USA). Labelled amplicons were purified by gel filtration through Sephadex G-50 (GE Healthcare, UK) and analyzed with the ABI PRISM 3730 Genetic Analyzer (Applied Biosystems Inc., USA). Multiple alignment of *CaM* sequences, and similarity score against reference sequences were performed by Bionumerics 5.1 software (Applied Maths, Belgium), determining the best-scoring reference sequence of the similarity output (≥99% against references sequences).

### Mycotoxins analysis

Fumonisin B_2_ (FB_2_) and OTA production capabilities were assessed according to Frisvad et al. (2007) for each strain, on CY20S and YES agar media, respectively, as described below. *In vitro* production of OTA and FB_2_ was examined on a subset of 88 and 95 isolates, respectively, including species-reference strains. For FB_2_ analysis, each CY20S agar cultures (1 g) was extracted with 5 ml of methanol/water (70:30, v/v) on orbital shaker for 60 min. One hundred microliter were diluted with 900 μl of acetonitrile/water (30:70, v/v) and then filtered using RC 0.2 μm filters (Phenomenex, USA). Fifty microliter of the extract were derivatized with 50 μL of o-phtaldialdehyde (OPA) and mixed for 50 s using an Agilent 1100 HPLC autosampler (Agilent Technologies, USA). One hundred microliter of the resulting derivatized samples were injected by full loop at 3 min after adding the OPA reagent for fumonisin analysis. The analytical column was a SymmetryShield RP18 (15 cm × 4.6 mm i.d., 5 μm) with a guard column inlet filter (0.5 μm × 3 mm i.d.) (Rheodyne, Idex Corporation, USA). The mobile phase consisted of 57% of solvent A (water/acetic acid, 99:1, v/v) and 43% of solvent B (acetonitrile/acetic acid, 99:1, v/v). The initial composition was kept constant for 5 min, then solvent B was linearly increased to 54% over 21 min, then up to 58% at 25 min and kept constant for 5 min. The column temperature was 30°C. The flow rate of the mobile phase was 0.8 ml/min. The fluorometric detector was set at wavelengths 335 nm for excitation and 440 nm for emission. FB_2_ was quantified by measuring peak areas, and comparing them to a calibration curve obtained with standard solutions. The detection limit for FB_2_ was 0.1 μg/g based on a signal-to-noise ratio of 3:1.

For extraction and detection of OTA, YES agar (1 g) cultures were extracted with 5 ml of methanol/water (70:30, v/v) on an orbital shaker for 60 min. One hundred microliter were diluted with 900 μl of acetonitrile/water (30:70, v/v) and then filtered using RC 0.2 μm filters (Phenomenex, USA). Fifty microliter of the extract were injected into an Agilent 1100 HPLC autosampler (Agilent Technologies, USA) with a full loop injection system. The analytical column was a Zorbax SB-C18 (4.6 mm × 150 mm i.d., 5 μm) with a guard column inlet filter (0.5 μm × 3 mm i.d.) (Rheodyne, Idex Corporation, USA). The mobile phase consisted of a mixture of acetonitrile/water/glacial acetic acid (99:99:2, v/v/v) at a flow rate of 1 ml/min. The fluorometric detector was set at 340 nm for excitation and 460 nm for emission. Ochratoxin A was measured by comparing peak areas with a calibration curve obtained with OTA standard solutions (Sigma-Aldrich, USA). The detection limit for OTA was 5 μg/kg based on a signal-to-noise ratio of 3:1.

### PCR primers and amplification conditions

The presence/absence of 10 genes and 3 intergenic regions within the putative *fum* cluster was assessed by PCR-based approach using primers previously described by Susca et al. (2014a). The amplicons represented 13 regions along almost the entire length of the *FUM* cluster described in *A. niger* strain ATCC 1015 (GenBank accession ACJE00000000) and strain CBS513.88 (GenBank accessions AM269948; AM270415). Eleven of the amplicons corresponded to the following 10 *fum* genes (listed in their order along the chromosome): *fum1, fum15, fum21* (two amplicons), *fum14, fum13, fum8, fum3, fum7, fum10*, and *fum6*. In addition, one amplicon corresponded to a region downstream of *fum6* (*fum6* ds), and another amplicon corresponded to the *fum19*-*fum15* intergenic region (*fum19-15* IGR). The presence and absence of four genes within the *ota* cluster was also assessed by a PCR-based approach using four primer sets. One primer set (pks15ksF/R) targeted the polyketide synthase gene *ota1* and was previously described (Ferracin et al., 2012). The three other primer sets were designed during the course of the current study and are based on genomic DNA sequence of *A. niger* strain CBS 513.88, which was available from the GenBank database of the National Center for Biotechnology Information (NCBI). Each of the three latter primer sets were designed to amplify a fragment of a different *ota* gene: *ota2, ota3*, and *ota5*. The primer pairs were designed using Primer Express® 2.0 software (Applied Biosystems Inc., USA). Table 1 summarizes the target genes, accession numbers, predicted functions, sequences of and amplicon sizes for each of the primer sets designed to amplify *fum* and *ota* gene fragments.

**Table 1:** **Information on target genes/regions and primers used for PCR in this study**.

	**Gene/Intergenic region name**	**Gene locus tag**	**Accession number**	**Predicted gene function**	**Primer sequences (5g′-3′)**	**Amplicon size**	**References**
*fum* cluster	downstream *fum6*	–	NT_166518.1	Intergenic region	f: CAAAAGACACCGCCCGTCT r: TTGACGCCCTGTACAAGGC	667 bp	Susca et al., 2014a
	*fum6*	ANI_1_2654014	NT_166518.1	NADPH-cyt P450 reductase	f: CTGTGAGGCCCTGGCACTT r: TCTGCCGGAGCTCAACGTA	849 bp	Susca et al., 2014a
	*fum10*	ANI_1_2658014	NT_166518.1	Peroxisomal-coenzyme A synthetase	f: GTCATTATTCCTCCGGCCCT r: TGGGATTCGAAAGCATACCG	651 bp	Susca et al., 2014a
	*fum7*	ANI_1_2660014	NT_166518.1	Fe-containing alcohol dehydrogenase	f: CAACAGCCCGAATCCCAGTA r: GCTCAGTCTTGCCCATCGTG	681 bp	Susca et al., 2014a
	*fum3*	ANI_1_892014	NT_166518.1	Dioxygenase	f: TACCATGGACCACTTTCCCG r: AAGTTCCTCAAGCGGCAGTC	651 bp	Susca et al., 2014a
	*fum8*	ANI_1_894014	NT_166518.1	a-oxoamine synthase	f: TTCGTTTGAGTGGTGGCA r: CAACTCCATASTTCWWGRRAGCCT	651 bp	Susca et al., 2014a
	*fum13*	ANI_1_2662014	NT_166518.1	short chain dehydrogenase	f: ATGCTCTTCACCTCCTCCGG r: CACTCAACGAGGAGCCTTCG	651 bp	Susca et al., 2014a
	*fum14*	ANI_1_2664014	NT_166518.1	NRPS-like condensation domain	f: TTGGGCTGATGTGCTCTGTC r: CCTCGTAGACGTAATTGAGTAGTCCT	730 bp	Susca et al., 2014a
	*fum21* region I	–	NT_166518.1	Zn(II)2Cys6 DNA-binding protein	f: CATTTCATGGGACCTCAGCC r: AAGCACAGGTTCCGAATTTGA	703 bp	Susca et al., 2014a
	*fum21* region II	–	NT_166518.1	Zn(II)2Cys6 DNA-binding protein	f: GGGTCCCATTGCCTCAATT r: CAATGGAGTCGACGGTGTCAC	705 bp	Susca et al., 2014a
	*fum15*	ANI_1_2668014	NT_166518.1	Cytochrome P450 monoxygenase	f: CGATTGGTAGCCCGAGGAA r: CTTGATATTGCGGAGTGGTCC	701 bp	Susca et al., 2014a
	*fum 19-15 IGR*	–	NT_166518.1	Intergenic region	f: ACACCGCGAGAATTCCATG r: GCAGGCTGGTAGTAGCGACAT	868 bp	Susca et al., 2014a
	*fum1*	ANI_1_2672014	NT_166518.1	Polyketide synthase	f: GGGTTCCAGGCAGAATCGTAC r: GAACTCACATCCTTTTGGGCC	701 bp	Susca et al., 2014a
*ota* cluster	*ota1*	ANI_1_1836134	NT_166530.1	Polyketide synthase	f: CAATGCCGTCCAACCGTATG r: CCTTCGCCTCGCCCGTAG	776 bp	Ferracin et al., 2012
	*ota2*	ANI_1_1832134	NT_166530.1	Nonribosomal peptide-synthetase	f: GGGAAYRCTGAYGTCGTGTTT r: TCCCACGAGCAWACAGCCTC	644 bp	This study
	*ota3*	ANI_1_1830134	NT_166530.1	Cytochrome P450	f: TTAGACAAACTGCGCGAGGA r: GCGTCGCTATGCCCAGATA	613 bp	This study
	*ota5*	ANI_1_1826134	NT_166530.1	radH flavin-dependent halogenase	f: TCCCTCGGTAAGAGTATCCTCGT r: GCGAGTTCTTGGTTCATGACG	845 bp	This study
	*CaM*	ANI_1_1116184	NT_166539.1	Calmodulin	f: GARTWCAAGGAGGCCTTCTC r: TTTTTGCATCATGAGTTGGAC	650 bp	O'Donnell et al., 2000

*The locus tags and accession numbers are GenBank/NCBI gene and contig designations, respectively, for A. niger strain CBS 513.88. Predicted gene functions are based on sequence homology to genes with known functions present in the GenBank/NCBI database*.

PCR reactions were carried out in a 10 μl volume containing: 0.25 U of HotMaster™ Taq DNA Polymerase (5 Prime, Germany), 1x HotMaster™ Taq DNA Polymerase buffer, 300 nM each primer, 200 μM deoxynucleotide mix (5 Prime, Germany) and approximately 20 ng of fungal DNA template. The PCR amplification program for *fum* genes was the same for the 13 primer sets, and was as follows: denaturation at 95°C for 2 min; 35 cycles of denaturation at 94°C for 50 s, annealing at 58°C for 50 s, extension at 72°C for 50 s; final extension at 72°C for 7 min, followed by cooling at 4°C until samples recovery. The PCR amplification program for genes in the *ota* cluster was the same for the 4 primer sets, and was as follows: denaturation at 95°C for 2 min; 35 cycles of denaturation at 94°C for 30 s, annealing at 60°C for 30 s, extension at 72°C for 30 s; final extension at 72°C for 7 min, followed by cooling at 4°C until samples recovery. Primer annealing temperature (60°C) for 3 OTA primer sets, designed in the present study, was empirically determined through gradient analysis.

Amplification of the approximately 650-bp fragment of the calmodulin gene, using CL1/CL2A primer (O'Donnell et al., 2000), was used as internal control for PCR assays. *A. niger* CBS 513.88 (ITEM 9568) was used as reference strain for all PCR experiments, because its genome sequence includes all *fum* and *ota* genes (Pel et al., 2007; Andersen et al., 2011). PCR products were electrophoresed in a 1.0% w/v agarose gel, stained with GelRed™ (Biotium Inc., USA) and visualized under UV transillumination.

### Examination of *fum* and *ota* cluster regions in genome sequences

*fum* and *ota* cluster regions in strains of *A. niger* and *A. welwitschiae* were retrieved from newly and previously generated genome sequences of these fungi. The previously generated sequences were from four strains of *A. niger* (ATCC 1015, ATCC 13496, CBS 513.88, and ITEM 10355) and three strains of *A. welwitschiae* (ATCC 13157, ITEM 7468, ITEM 11945). The ATCC 1015, ATCC 13157, and ATCC 13496 sequences were examined using the Joint Genome Institute (JGI) Genome Portal website (http://genome.jgi.doe.gov/), and the CBS 513.88 sequence was examined using the NCBI website (http://www.ncbi.nlm.nih.gov/). The genome sequences for strains ITEM 7468, ITEM 10355, and ITEM 11945 were previously generated by Susca et al. (2014a).

In order to examine the *fum* and *ota* cluster regions in selected isolates of *A. welwitschiae*, whole genome sequences were generated for the following isolates: ITEM 4552, ITEM 6142, ITEM 6144, ITEM 7097, ITEM 10353, ITEM 10929, ITEM 10932, ITEM 11209, ITEM 11980, ITEM 11984, ITEM 14309, ITEM 15179, ITEM 15309. Each isolate was grown in YEDP medium (0.1 yeast extract, 0.1 peptone, 2% dextrose) for 2 days at room temperature with shaking at 200 rpm. Mycelia were harvested by filtration, lyophilized, ground to a powder, and genomic DNA was extracted using the method described by Raeder and Broda (1985). The resulting DNA was further purified with the UltraClean DNA purification kit according to the specifications of the manufacturer (MoBio Laboratories, Inc.). DNA libraries were prepared using a NExtera XT DNA library Preparation Kit, and sequence data were generated with an Illumina MiSeq sequencing platform as specified by the manufacturer (Illumina, San Diego, California). CLC Genomics Workbench (CLC bio, Qiagen, Aarhus, Denmark) was used to process the resulting sequence reads and obtain a *de novo* assembly of each genome.

For ITEM isolates, sequences of the *fum* and *ota* cluster regions were retrieved from the genome sequences via the BLASTn (Altschul et al., 1997) function in CLC Genomics Workbench and using *A. niger* homologs of *fum, ota*, and cluster flanking genes, which have been previously reported (Pel et al., 2007; Gallo et al., 2012, 2014; Susca et al., 2014a). Sequences corresponding to the *fum* and *ota* cluster regions from strains have been deposited into the GenBank database at NCBI as accessions KX267735–KX267737. Phylogenetic analysis of homologous sequences from the *ota* cluster region was done using the phylogenetic analysis software MEGA 5 (Tamura et al., 2011). The sequences were aligned using Muscle, and the resulting alignment was subjected to a model selection analysis to determine the best nucleotide substitution model and to maximum likelihood analysis.

## Results

### Fungal identification

The species identity of the 92 black aspergilli isolates, which had *A. niger* morphology in culture based on characteristic conidial morphology (Kozakiewicz, 1989), was determined by comparison of the DNA sequence of a PCR-amplified *caM* fragment from each isolate to the *caM* sequences for reference strains of *A. niger* and *A. welwitschiae*. Previous analyses have shown that the *caM* sequence can be used to distinguish between *A. niger* and *A. welwitschiae* (Perrone et al., 2011). The comparisons revealed that sequences from 67 isolates were 99.75–100.00% identical to the reference sequence from *A. welwitschiae* strains ITEM 4509 and ITEM 4552, and sequences from 25 isolates was 99.75–100.00% identical to the reference sequence from *A. niger* strain ITEM 4501. Previous phylogenetic analyses of some of the isolates employed in this study indicate that such differences in sequence identity are reliable indicators of the species identity of *A. niger* and *A. welwitschiae* (Susca et al., 2014a,b). Based on this, we concluded that 67 isolates in the collection were *A. welwitschiae*, and 25 were *A. niger*.

The sample sizes of isolates from almonds, cashews, maize, pistachios and walnuts ranged from two to eight, making it difficult to evaluate whether one species occurred more frequently than the other on these crops. However, the sample sizes from grapes and raisins were 33 and 34 respectively, and *A. welwitschiae* occurred more frequently on both; 82% of the isolates from these two crops were *A. welwitschiae*, and 18% were *A. niger*.

### Mycotoxin production

The levels of FB_2_ and OTA produced by the collection of *A. niger* and *A. welwitschiae* isolates varied markedly. Among producing isolates, the levels of OTA produced (30–590 μg/kg) were typically higher than the levels of FB_2_ (0.1–44.4 μg/g). The frequency of FB_2_-producing and nonproducing isolates was markedly different in the two species. That is, a majority of *A. niger* isolates (74%) produced FB_2_, but a minority of *A. welwitschiae* isolates (16%) produced FB_2_ (Table 2). In contrast, a minority of isolates of both species produced OTA: 4% of *A. niger* isolates and 25% of *A. welwitschiae* isolates. The frequency of isolates that produced both mycotoxins was similar in the two species: 4% of *A. niger* isolates and 8% of *A. welwitschiae* isolates (Table 2). In contrast, the frequency of isolates that produced neither mycotoxin differed markedly: 13% of *A. niger* isolates and 68% of *A. welwitschiae* isolates (Table 2).

**Table 2:** **Strain designations, crop and geographic origins, FB_**2**_ and OTA production, and mycotoxin biosynthetic gene amplification patterns for ***A. niger*** and ***A. welwitschiae*** isolates examined in this study**.

**ITEM**	**Species**	**Substrate**	**Origin**	**FB_2_ (μg/g)**	***fum* amplicon pattern**	**OTA (μg/kg)**	***ota* amplicon pattern**
**4501**	*A. niger*			0.4	f-1	−	o-1
**9568**	*A. niger*			+	f-1	+	o-1
11461	*A. niger*	Almonds	USA	6.4	nd	−	o-2
11432	*A. niger*	Cashew nuts	Brazil	18.7	nd	−	o-2
11433	*A. niger*	Cashew nuts	Brazil	36.7	nd	−	o-2
11446	*A. niger*	Cashew nuts	Brazil	41.7	nd	−	o-2
11449	*A. niger*	Cashew nuts	Brazil	40.4	nd	−	o-2
11451	*A. niger*	Cashew nuts	Brazil	29.1	nd	−	o-2
11558	*A. niger*	Cashew nuts	India	3.2	nd	−	o-2
5218	*A. niger*	Grape	Greece	−	f-1	−	o-2
5219	*A. niger*	Grape	Greece	−	f-1	−	o-2
5276	*A. niger*	Grape	Greece	0.6	f-1	−	o-2
7090	*A. niger*	Grape	Italy	−	f-1	−	o-2
7091	*A. niger*	Grape	Italy	3.3	f-1	−	o-2
10355	*A. niger*	Grape	Italy	−	f-1	−	o-2
15310	*A. niger*	Maize	USA	−	f-1	n.t.	nd
15342	*A. niger*	Maize	USA	−	f-1	n.t.	nd
15369	*A. niger*	Maize	USA	−	f-1	n.t.	nd
15374	*A. niger*	Maize	USA	0.1	f-1	n.t.	nd
12736	*A. niger*	Pistachio	Italia	9.5	nd	−	o-2
10939	*A. niger*	Pistachio	USA	4.3	nd	−	o-2
10924	*A. niger*	Raisins	Turkey	2.8	nd	−	o-2
10927	*A. niger*	Raisins	Turkey	10.7	nd	−	o-2
11930	*A. niger*	Raisins	Turkey	7.8	nd	−	o-2
11941	*A. niger*	Raisins	Turkey	1.1	nd	−	o-2
14275	*A. niger*	Raisins	Turkey	0.6	f-1	−	o-2
14281	*A. niger*	Raisins	Turkey	0.5	f-1	−	o-2
11196	*A. welwitschiae*	Almonds	Italy	−	f-13	−	o-2
11197	*A. welwitschiae*	Almonds	Italy	−	f-13	−	o-2
11435	*A. welwitschiae*	Cashew nuts	Brazil	19.2	f-1	−	o-2
11447	*A. welwitschiae*	Cashew nuts	Brazil	44.4	f-1	−	o-2
11774	*A. welwitschiae*	Grape	Argentina	−	f-13	−	o-2
11778	*A. welwitschiae*	Grape	Argentina	−	f-13	−	o-2
11786	*A. welwitschiae*	Grape	Argentina	−	f-13	−	o-2
11828	*A. welwitschiae*	Grape	Argentina	−	f-13	40.0	nd
11836	*A. welwitschiae*	Grape	Argentina	−	f-13	−	o-2
11844	*A. welwitschiae*	Grape	Argentina	−	f-13	−	o-2
11854	*A. welwitschiae*	Grape	Argentina	−	f-13	−	o-2
11867	*A. welwitschiae*	Grape	Argentina	−	f-13	−	o-2
5253	*A. welwitschiae*	Grape	Greece	−	f-13	−	o-2
5267	*A. welwitschiae*	Grape	Greece	−	f-13	−	o-2
5277	*A. welwitschiae*	Grape	Greece	10.9	f-1	−	o-2
4717	*A. welwitschiae*	Grape	Italy	−	f-13	−	o-2
4853	*A. welwitschiae*	Grape	Italy	−	f-13	−	o-2
4858	*A. welwitschiae*	Grape	Italy	−	f-13	−	o-2
4863	*A. welwitschiae*	Grape	Italy	−	f-13	−	o-2
6122	*A. welwitschiae*	Grape	Italy	−	f-13	−	nd
6126	*A. welwitschiae*	Grape	Italy	−	f-13	280.0	nd
6127	*A. welwitschiae*	Grape	Italy	−	f-12	−	nd
6128	*A. welwitschiae*	Grape	Italy	−	f-12	−	o-2
7097	*A. welwitschiae*	Grape	Italy	7.5	f-1	590.0	o-1
7468	*A. welwitschiae*	Grape	Italy	−	f-13	−	o-2
10353	*A. welwitschiae*	Grape	Italy	−	f-9	−	o-2
**4552**	*A. welwitschiae*	grape	Portugal	−	f-13	45.0	o-1
6140	*A. welwitschiae*	Grape	Portugal	−	f-7	−	o-2
6142	*A. welwitschiae*	Grape	Portugal	−	f-7	545.0	o-1
6144	*A. welwitschiae*	Grape	Portugal	−	f-9	380.0	nd
4947	*A. welwitschiae*	Grape	Spain	−	f-13	−	o-2
15095	*A. welwitschiae*	Maize	Italy	0.1	f-1	n.t.	nd
15179	*A. welwitschiae*	Maize	Italy	−	f-3	n.t.	nd
15309	*A. welwitschiae*	Maize	USA	−	f-2	n.t.	nd
13288	*A. welwitschiae*	Pistachio	Iran	−	f-13	−	o-2
11734	*A. welwitschiae*	Pistachio	USA	−	f-13	−	o-2
12780	*A. welwitschiae*	Pistachio	USA	−	f-12	−	o-2
10635	*A. welwitschiae*	Raisins	Turkey	−	f-12	−	o-2
10636	*A. welwitschiae*	Raisins	Turkey	−	f-13	−	o-2
10637	*A. welwitschiae*	Raisins	Turkey	−	f-12	−	o-2
10929	*A. welwitschiae*	Raisins	Turkey	−	f-4	−	nd
10932	*A. welwitschiae*	Raisins	Turkey	−	f-11	−	o-2
10935	*A. welwitschiae*	Raisins	Turkey	2.9	f-1	−	o-2
11925	*A. welwitschiae*	Raisins	Turkey	−	f-13	−	o-2
11931	*A. welwitschiae*	Raisins	Turkey	−	f-11	−	o-2
11943	*A. welwitschiae*	Raisins	Turkey	−	f-13	−	o-2
11945	*A. welwitschiae*	Raisins	Turkey	25.3	f-1	−	o-2
11948	*A. welwitschiae*	Raisins	Turkey	−	f-12	30.0	nd
11980	*A. welwitschiae*	Raisins	Turkey	−	f-10	50.0	nd
11982	*A. welwitschiae*	Raisins	Turkey	−	f-13	−	o-2
11984	*A. welwitschiae*	Raisins	Turkey	−	f-12	55.0	nd
11990	*A. welwitschiae*	Raisins	Turkey	−	f-12	−	o-2
12113	*A. welwitschiae*	Raisins	Turkey	−	f-12	−	o-2
12115	*A. welwitschiae*	Raisins	Turkey	−	f-13	40.0	nd
12246	*A. welwitschiae*	Raisins	Turkey	−	f-13	−	nd
12591	*A. welwitschiae*	Raisins	Turkey	−	f-5	110.0	nd
12594	*A. welwitschiae*	Raisins	Turkey	−	f-13	−	o-2
14297	*A. welwitschiae*	Raisins	Turkey	9.5	f-1	35.0	nd
14303	*A. welwitschiae*	Raisins	Turkey	3.5	f-1	45.0	nd
14305	*A. welwitschiae*	Raisins	Turkey	3.6	f-1	45.0	nd
14307	*A. welwitschiae*	Raisins	Turkey	−	f-13	−	nd
14308	*A. welwitschiae*	Raisins	Turkey	1.0	f-1	40.0	nd
14309	*A. welwitschiae*	Raisins	Turkey	−	f-8	45.0	nd
14310	*A. welwitschiae*	Raisins	Turkey	−	f-13	−	nd
12120	*A. welwitschiae*	Raisins	USA	−	f-13	−	o-2
10908	*A. welwitschiae*	Walnuts	Chile	−	f-13	−	o-2
10910	*A. welwitschiae*	Walnuts	Chile	−	f-13	−	o-2
11209	*A. welwitschiae*	Walnuts	China	−	f-6	−	o-2

*−: Not detected with the limit of detection (LOD): 5 μg/kg (OTA), 0.1 μg/g (FB_2_). nt: not tested; nd: not determined. bold type: species reference strain. +: strain reported in literature as mycotoxin producer (Andersen et al., 2011)*.

**Figure 2:**
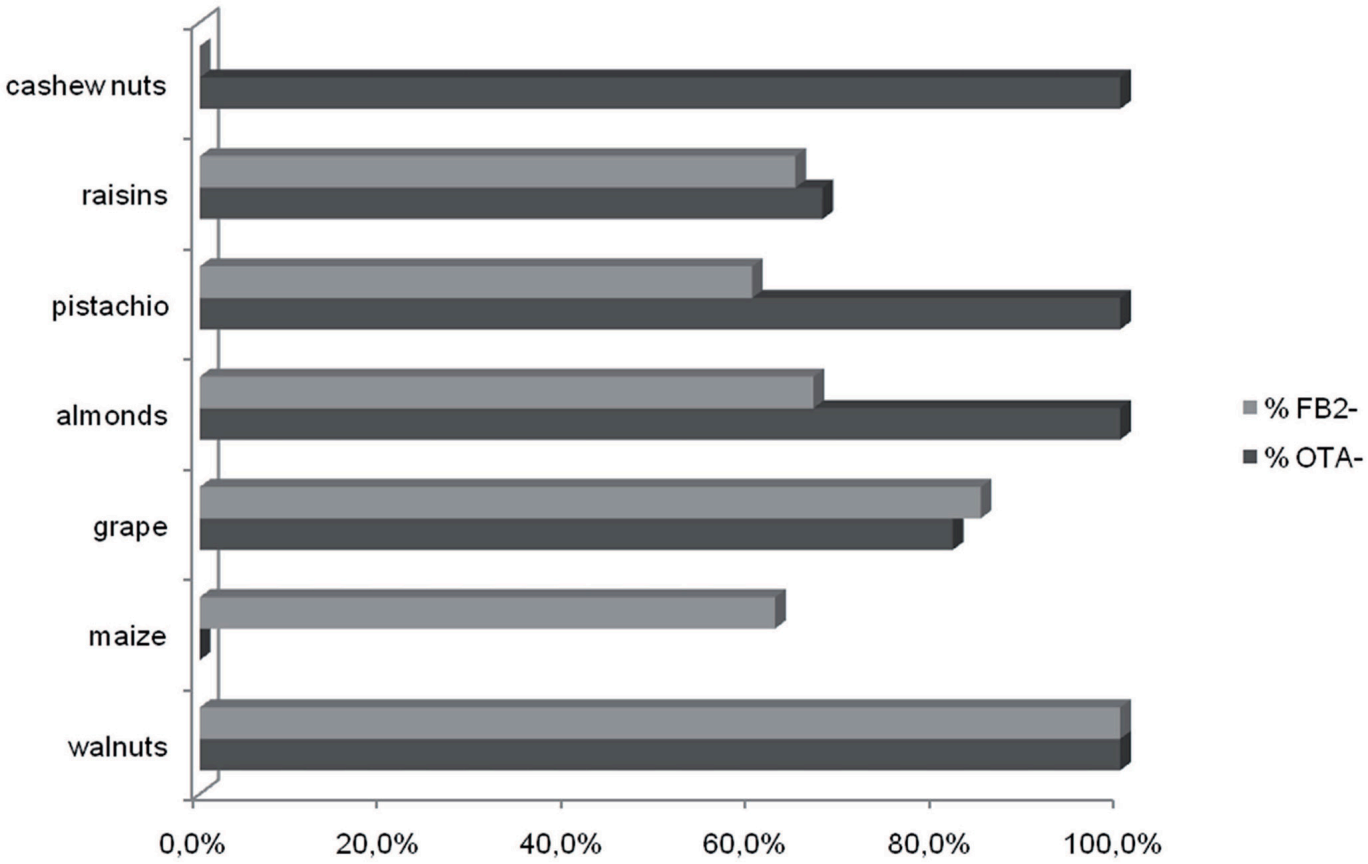
**Occurrence of FB_**2**_- and OTA-nonproducing isolates on different crops**. Values are percentages for each crop.

**Figure 3:**
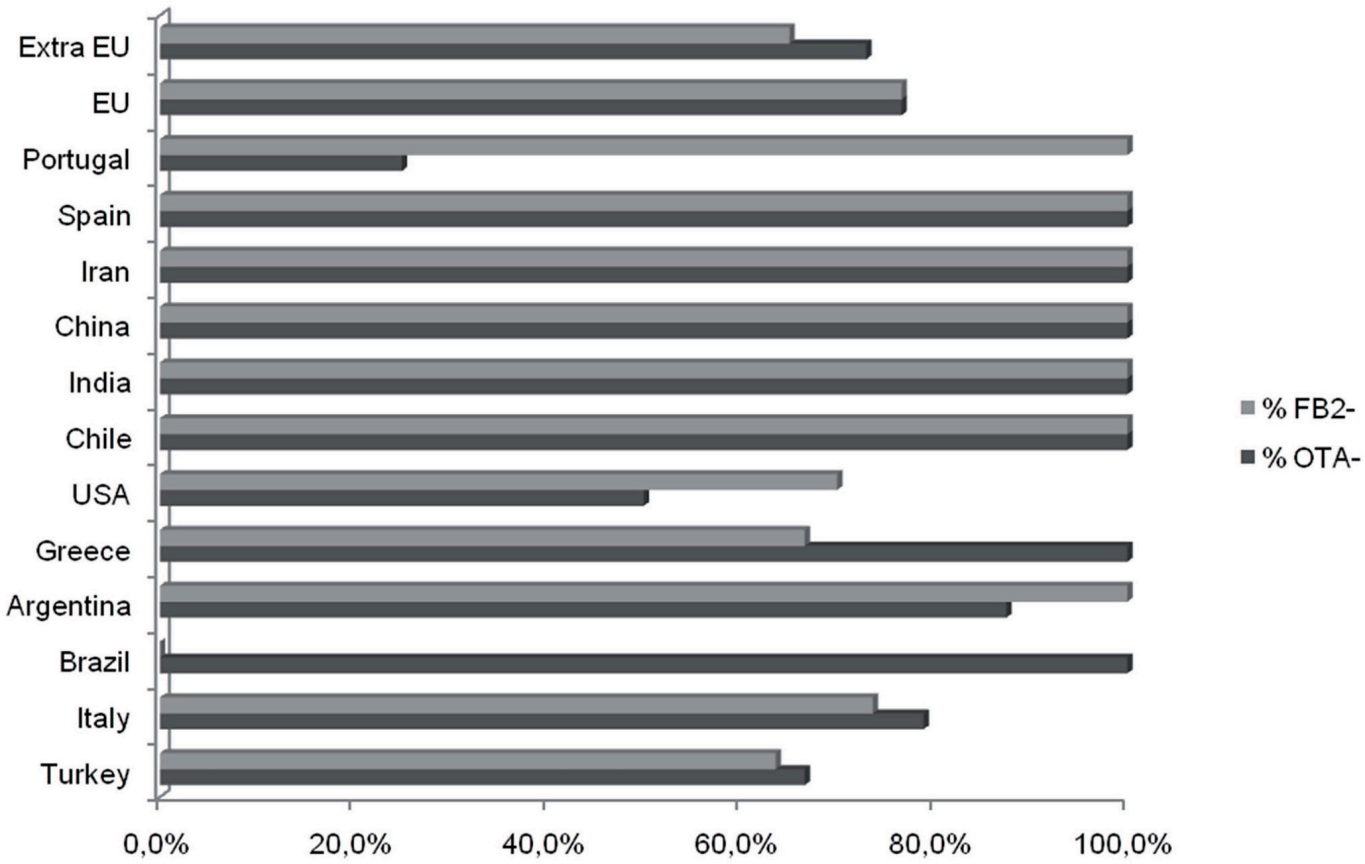
**Occurrence FB_**2**_- and OTA-nonproducing isolates from different geographic origins**. Values are percentages for each Country.

Although the sample sizes for sets of isolates recovered from a given crop were small, the data indicate some possible differences among isolates from different crops (Figure 2). As noted above, the majority of *A. niger* isolates produced FB_2_. However, all *A. niger* isolates from cashews and raisins produced FB_2_, whereas most isolates from grape and maize did not (Table 2). Although most isolates of *A. welwitschiae* did not produce FB_2_ or OTA, there was a relatively high proportion of FB_2_ and OTA-producing isolates from raisins: 6 of 27 isolates produced FB_2_, and 10 of 27 isolates produced OTA. In addition, 4 of the 5 *A. welwitschiae* isolates that produced both mycotoxins originated on raisins. OTA-nonproducing isolates occurred with almost equal frequencies in European (77%) and non-European (73%) regions, while FB_2_-nonproducing isolates occurred slightly more frequently in European (77%) than in non-European (65%) locations (Figure 3).

### Variation in mycotoxin biosynthetic gene content

#### *fum* cluster genes

**Figure 4:**
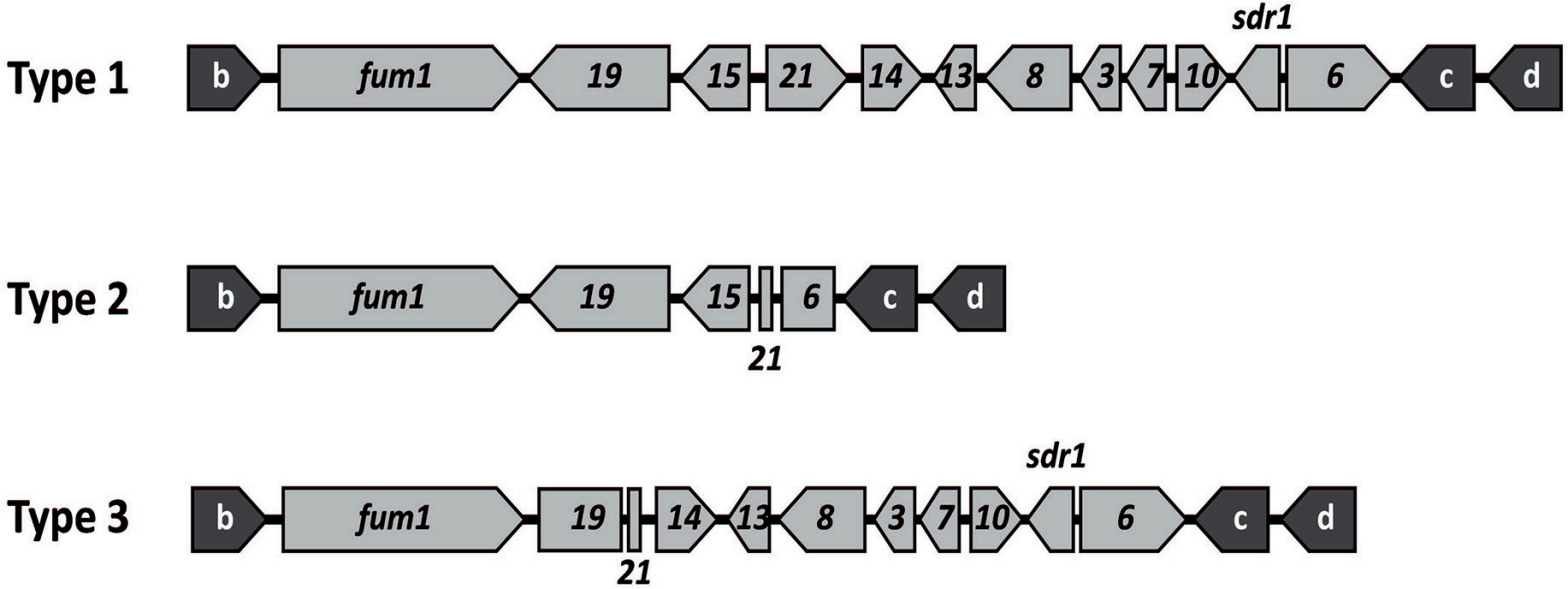
**Three ***fum*** cluster types observed in newly generated genome sequences of isolates of ***A. welwitschiae*****. Type 1, an intact cluster, was observed in isolates ITEM 7097, 11209, and 15309. Type 2, a partially deleted cluster, was observed in isolates ITEM 4552, 6142, 10353, 10932, 11209, 11980, 11984, 14309, and 15309. Type 3, also a partially deleted cluster, was observed in isolate ITEM 10929 only. The gene content and arrangement in Type 1 is identical to the *fum* cluster previously described in the FB-producing *A. welwitschiae* isolate ITEM 11945 as well as all *A. niger* isolates examined; and the gene content and arrangement in Type 2 is identical to the *fum* cluster previously described in *A. welwitschiae* isolate ITEM 7468 (Susca et al., 2014a). As far as we are aware, Type 3 has not been described previously. Gene designations are as in Figure 1. Genes shown as arrows have full-length coding regions and are most likely functional, whereas, genes shown as rectangles/squares are truncated and therefore nonfunctional. Genes labeled b, c, and d are previously described *fum* cluster flanking genes (Susca et al., 2014a).

The presence and absence of the 10 genes in the *fum* cluster were assessed initially by the PCR assay described previously (Susca et al., 2014a) using the primers shown in Table 1. The seven FB_2_-producing and 7 FB_2_-nonproducing isolates of *A. niger* examined yielded amplicons for all *fum* genes and intergenic regions examined. These PCR results indicated that there were no apparent differences in the gene content of the *fum* cluster of FB-producing and nonproducing isolates of *A. niger*, a finding that is consistent with previous analyses of the cluster in this species (Palumbo et al., 2013; Susca et al., 2014a). A larger set of 8 FB_2_-producing and 57 nonproducing isolates of *A. welwitschiae* was also subjected to the *fum*-gene PCR assay. The analysis yielded 13 amplicon patterns: the two patterns previously described for FB-producing and nonproducing isolates of the species (Susca et al., 2014a) as well as 11 novel patterns. In Table 2, the previously described amplification patterns for FB-producing and nonproducing isolates of *A. welwitschiae* were designated as Patterns f-1 and f-2, respectively, while the 11 novel amplification patterns were designated as Patterns f-3 through f-13. To determine whether the novel amplification patterns correspond to undescribed deletions within the *fum* cluster, we generated whole-genome sequence data for isolates of *A. welwitschiae* representative of the novel patterns, except f-5. Retrieval and analysis of sequences of the *fum* cluster region from each genome sequence revealed that, in all isolates examined except ITEM 10929, the gene content of the *fum* cluster in isolates with the novel PCR amplification patterns was identical to one of the two *fum* cluster types previously described for *A. welwitschiae* (Susca et al., 2014a), and designated as cluster Types 1 and 2 in Figure 4. In Type 1, there were full-length homologs of all 11 *fum* genes and the *sdr1* gene. In Type 2, six *fum* genes and *sdr1* were absent, and *fum6* and *fum21* were truncated, whereas, *fum1, fum15*, and *fum19* were intact. Thus, the novel *fum* gene amplification patterns observed in the PCR assay were not consistent with the genomic sequences.

The analysis of the genome sequences of *A. welwitschiae* isolates ITEM 11209 and ITEM 15309 revealed that the *fum* cluster homologs in these two strains contained all 11 *fum* genes as well as *sdr1*. As far as we are aware, this is the first report of a full-length *fum* clusters in FB-nonproducing isolates of *A. welwitschiae*. In ITEM 15309, all *fum* genes appeared to be functional in that there were no insertions or deletions that would result in frameshifts or premature stop codons in the gene coding regions of any of the genes. Thus, the sequence of the *fum* cluster in this strain did not provide clues as to the genetic basis for the FB-nonproduction phenotype of the isolate. In ITEM 11209, all the *fum* genes appeared to be functional except for *fum1*, which had a C-to-T transition that changed codon 2163 from CGA to TGA, a stop codon. This premature stop codon would most likely render the *fum1*-encoded polyketide synthase nonfunctional, because it would block translation of the mRNA before synthesis of keto reductase and phosphopantetheine domains, two domains that are essential for function of the polyketide synthase. As noted above, isolate ITEM 10929 was an exception; it had a *fum* cluster in which *fum19* and *fum21* were truncated, *fum15* was absent, but the other eight *fum* genes and *sdr1* were intact and apparently functional (Figure 4). Thus, the *fum* cluster in ITEM 10929 differed from the two previously described cluster types in *A. welwitschiae*.

#### OTA cluster genes

The presence and absence of four *ota* cluster genes (*ota1*-*ota3* and *ota5*) was also assessed in a subset of 69 isolates using a PCR-based assay. There were only two patterns of amplicons observed. The first pattern (Pattern o-1) consisted of amplicons for the four *ota* cluster genes examined. All OTA-producing isolates of both species (*A. niger* ITEM 9568 and *A. welwitschiae* ITEM 7097, ITEM 6142, ITEM 4552) exhibited Pattern o-1. The amplicon sizes were consistent with the expected sizes based on the design of PCR primers: 776 bp for *ota1*, 645 bp for *ota2*, 614 bp for *ota3*, and 846 bp for *ota5*. In the second *ota* PCR pattern (Pattern o-2) none of the amplicons was present. All OTA-nonproducing isolates of both *A. niger* (21 isolates) and *A. welwitschiae* (44 isolates) exhibited Pattern o-2 (Table 2). The absence of amplicons for the 4 putative *ota* cluster genes is consistent with the previously described 21-Kb deletion in the *ota* cluster region of the *A. niger* OTA-nonproducing strain ATCC 1015 (Andersen et al., 2011).

**Figure 5:**
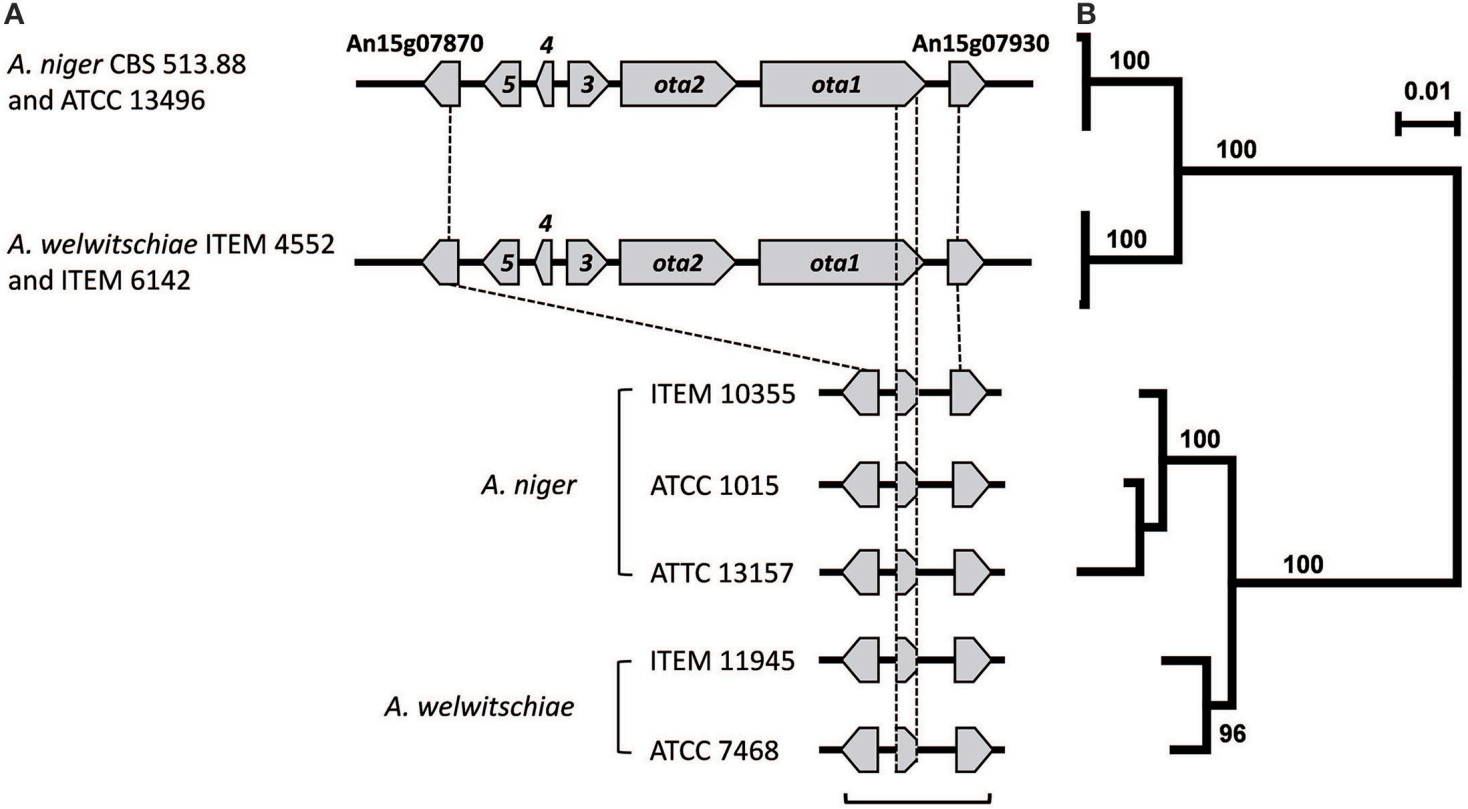
**(A)** Comparison of the intact and deleted *ota* cluster region in selected isolates of *A. niger* and *A. welwitschiae*. Arrows and gene designations are as in Figure 1. An15g07870 and An15g07930 are genes flanking each side of the *ota* cluster; and the An15g07870 and An15g07930 designations are the original gene model designations for GenBank accessions for the genome sequence of *A. niger* strain CBS 513.88. **(B)** Maximum likelihood tree inferred from the An15g07870-An15g07930 region: i.e., the region spanned by the bracket at the bottom of **(A)** in strains that lack an *ota* cluster as well as the homologous sequences from strains that have an intact cluster. The scale at the upper right indicates the number of substitutions per site. On the JGI website, ATCC 13157 is classified as *A. phoenicis*. Although *A. phoenicis* is considered to be synonymous with *A. welwitschiae* (Hong et al., 2013), our analysis indicates that ATCC 13157 is a strain of *A. niger sensu stricto* (Figure S1).

To further evaluate whether the amplification Patterns o-1 and o-2 were consistent with the presence and absence of *ota* genes, we examined the genome sequences from a subset of one *A. niger* and seven *A. welwitschiae* isolates for which amplification-pattern data were determined. This analysis revealed that the three *A. welwitschiae* isolates (ITEM 4552, ITEM 6142, and ITEM 7097) that exhibited Pattern o-1 had an intact *ota* cluster; i.e., they had all five *ota* genes, and the genes were apparently functional. Further, the sequence analysis indicated that the one *A. niger* isolate (ITEM 10355) and the four *A. welwitschiae* isolates (ITEM 7468, ITEM 10353, ITEM 10932, and ITEM 11209) with Pattern o-2 did not have any of the *ota* genes, except for an approximately 1100-nucleotide region near the 3′ end of *ota1*. These results indicate that the *ota* amplification patterns were consistent with the presence and absence of *ota* genes. Furthermore, the results of both the PCR and genome sequence data provide evidence that the OTA-nonproduction phenotype in the majority of isolates of both *A. niger* and *A. welwitschiae* results from the absence of *ota* genes in the isolates. Examination of previously generated genome sequences for the *ota* cluster confirmed that the cluster was intact in *A. niger* strain CBS 513.88 and absent in *A. niger* strain ATCC 1015 as previously reported (Andersen et al., 2011). This analysis also revealed that an intact *ota* cluster was present in *A. niger* strains ATCC 13157 and ATCC 13496, but not in *A. niger* strain ITEM 10355 as well as *A. welwitschiae* strains ITEM 7468 and ITEM 11945 (Figure 5A). Further examination of both previously generated genome sequence data and sequence data generated during this study (20 genome sequences in total) revealed that all strains examined have homologs of the *ota* cluster flanking genes An15g07870 and An15g07930 regardless of species and whether the strains have an intact cluster (Figure 5A). In both *A. niger* and *A. welwitschiae* sequences that lack the *ota* cluster, the An15g07870-An15g07930 intergenic region ranges in length from 2508 to 2800 nucleotides. 94% of this aligned intergenic sequence was homologous to three sequence elements in *A. niger* and *A. welwitschiae* strains with an intact *ota* cluster: 18% of the region was homologous to the sequence immediately 5′ to the An15g07870 start codon; 41% was homologous to sequence near the 3′ end of *ota1*; and 35% was homologous to sequence immediately 5′ to the An15g07930 start codon (Figure 5A). The identity of homologs of the three sequence elements ranged from 79 to 92% in strains with an intact vs. a deleted *ota* cluster. In strains that had a deleted cluster, the sequence corresponding to *ota1* was 1127–1178 nucleotides in length and was homologous to nucleotides 7012–8126 of the intact *ota1* coding region (with introns included). Variation in the length of the *ota1*-fragment homologs resulted from deletions and insertions within the homologs. Regardless of species, the *ota1*-fragment homologs began and ended at the same positions, nucleotides 7012 and 8126 respectively, of the intact *ota1* coding region. The homology of the three sequence elements within the An15g07870-An15g07930 intergenic region facilitated alignment and phylogenetic analysis of the sequences. The tree inferred from the analysis indicated that, regardless of species, sequences from strains with the *ota* cluster deletion are more closely related to one another than to sequences from strains with the intact *ota* cluster (Figure 5B).

## Discussion

The black aspergilli *A. niger* and *A. welwitschiae* are used in fermentation of food and beverages. However, concerns about the safety of these fungi have been raised with the discovery that some isolates can produce the mycotoxins FBs and OTA (Frisvad et al., 2007, 2011; Susca et al., 2014b). As a result, multiple studies have been initiated to investigate the occurrence and distribution of FB and OTA production as well as the genetic basis for nonproduction in both industrial and field strains of these fungi. The studies indicate that both species exist as mixed populations of FB-producing or FB-nonproducing individuals as well as OTA-producing or OTA-nonproducing individuals. In addition, all possible combinations of FB/OTA production and nonproduction have been reported. That is, isolates that are FB-producing but OTA-nonproducing, FB-nonproducing but OTA-producing, FB-nonproducing and OTA-nonproducing, or FB-producing and OTA-producing have been reported. The FB-producing and OTA-producing phenotype has been reported less frequently than the other phenotypes: 15 of 175 isolates examined for both mycotoxins in a study by Massi et al. (2016), and six of 88 isolates examined for both mycotoxins in the current study (Table 2).

Published surveys of FB and OTA production in *A. niger* and *A. welwitschiae* generally do not indicate whether the collections of isolates examined represent random samples or biased selections of isolates. As a result, caution must be exercised when drawing conclusions about trends in production and nonproduction of isolates from multiple crops and geographic regions that appear to be evident from comparisons of the surveys. Nevertheless, a comparison of results of the current study and five previously reported studies indicate some trends. First, the results from all studies indicate that a majority of *A. niger* isolates examined can produce FBs (Table 3). In contrast, most of the studies indicate that only a minority of *A. welwitschiae* isolates can produce FBs (Table 3). The one exception to this is from a collection of *A. welwitschiae* isolates recovered from onion grown in Saudi Arabia (Gherbawy et al., 2015), where approximately half (48%) of the isolates produced FBs (Table 3). The studies also indicate that only a minority of both *A. niger* and *A. welwitschiae* isolates can produce OTA (Table 3). In previous studies, 0–1% of *A. welwitschiae* isolates produced OTA. In current study, by contrast, a substantially higher percentage (25%) of *A. welwitschiae* isolates produced OTA. Thus, it is possible that environmental factors such plant species, location, climate and/or agricultural practices can alter the frequency of FB- and OTA-producing isolates.

**Table 3:** **Comparison of results from this and five previous studies on production of FBs and OTA in field isolates of ***A. niger*** and ***A. welwitschiae*****.

	**Fumonisin analysis**		**Ochratoxin analysis**	
	**No. isolates^a^**	**Producers^b^(%)**	**No. isolates^a^**	**Producers^b^ (%)**
***A. NIGER***				
This Study	27	74	24	4
Massi et al., 2016	89	74	89	31
Qi et al., 2016	10	100	10	0
Susca et al., 2014a	19	63	nd	nd
Storari et al., 2012	41	76	41	7
***A. WELWITSCHIAE***				
This Study	68	16	65	25
Massi et al., 2016	86	34	86	1
Qi et al., 2016	109	26	109	0
Gherbawy et al., 2015	37	48	37	0
Susca et al., 2014a	35	29	nd	nd
Storari et al., 2012	27	37	27	0

a *Number of isolates of A. niger or A. welwitschiae examined in each study for fumonisin or ochratoxin production. nd indicated not determined*.

b *Percentage of isolates examined that produced fumonisin or ochratoxin. nd indicated not determined*.

In addition to the frequency of mycotoxin production phenotypes, the comparison of studies suggests that *A. niger* and *A. welwitschiae* can vary in their frequencies of occurrence. *A. welwitschiae* appears to occur more frequently than *A. niger* in multiple host/location combinations. This was the case for grape and raisin isolates in the current study, for grape isolates in Canada (Qi et al., 2016), and for onion isolates from Brazil (Massi et al., 2016) and Saudi Arabia (Gherbawy et al., 2015; Table 3). However, this trend does not appear to exist for all crops and locations. For example, the numbers of *A. niger* and *A. welwitschiae* isolates from Brazil nuts grown in Brazil were similar, and the number of *A. niger* isolates (28) from grapes in Brazil was approximately two times greater than the number of *A. welwitschiae* isolates.

Several previous studies on *A. niger* and *A. welwitschiae* have also provided evidence that the genetic basis for the FB-nonproduction phenotype differs in the two species: the *fum* cluster is partially deleted in FB-nonproducing isolates of *A. welwitschiae*, whereas, the *fum* cluster is intact in FB-nonproducing isolates of *A. niger*. In the current study, PCR results for all *A. niger* isolates were consistent with previous results, whereas, results for some FB-nonproducing isolates of *A. welwitschiae* were not. However, sequence analysis of selected nonproducing isolates of *A. welwitschiae* indicated that, in most cases, the isolates had either one of the two *fum* cluster types that were previously described (Figure 4; Susca et al., 2014a). Therefore, the PCR results did not always accurately reflect the gene content of the *fum* cluster in *A. welwitschiae*, a phenomenon that has been noted previously (Palumbo et al., 2013). In addition, we identified an isolate (ITEM 15309) that had an intact *fum* cluster, but did not produce FBs, a situation that has been reported previously for *A. niger* but not *A. welwitschiae* (Susca et al., 2014a). Because isolate ITEM 15309 has apparently functional *fum* genes, it is not clear why this isolate does not produce FBs. In contrast, FB-nonproducing isolate ITEM 11209 had a *fum* cluster with a point mutation in *fum1* but apparently functional homologs of other *fum* genes. Because the point mutation likely renders *fum1* nonfunctional, and because *fum1* is required for the first committed biochemical reaction in fumonisin biosynthesis (Proctor et al., 1999), the mutation would almost certainly block FB production in ITEM 11209. Thus, the nonfunctional *fum1* in ITEM 11209 provides a possible explanation for why the isolate does not produce FBs. Likewise, the truncation of *fum21* in the novel *fum* cluster type in *A. welwitschiae* isolate ITEM 10929 could explain the lack of FB production in this isolate, because analysis of the *fum21* homolog in *Fusarium* has demonstrated that it is required for expression of other *fum* cluster genes and, therefore, FB production (Brown et al., 2007).

The PCR and sequence analyses of *ota* genes were consistent and indicated that in OTA-nonproducing isolates of both *A. niger* and *A. welwitschiae* the *ota* cluster is almost completely deleted. These results contrast those of Massi et al. (2016), who used PCR to examine 146 OTA-nonproducing isolates of these species for the presence of *ota1* and *ota5*. Although Massi et al. did not detect either gene in the majority of nonproducing isolates examined, they did detect the two genes in one nonproducing isolate of *A. niger* and six nonproducing isolates of *A. welwitschiae*. These findings could be an indication that factors other than the absence of *ota* genes contribute to the lack of OTA production. Alternatively, the findings could be an indication that multiple patterns of *ota* gene deletion exist among OTA-nonproducing isolates of *A. niger* and *A. welwitschiae*.

Regardless of the difference in the *ota*-based PCR results reported in the current study and the study by Massi et al. (2016), genome sequence data indicate deletion of DNA within the *ota* cluster region is almost identical in OTA-nonproducing strains of *A. niger* and *A. welwitschiae*. We propose two alternative scenarios to explain this observation. In the first scenario, deletion of the *ota* cluster resulted from independent events in the two species: one event (or series of events) in *A. niger* and another independent event (or series of events) in *A. welwitschiae*. In this scenario, however, the deletion events were nonrandom and left almost identical sequence elements, including an *ota1* fragment, in the An15g07870-An15g07930 intergenic region. In the second scenario, the intact and deleted *ota* clusters in the two species are descendants of ancestral alleles. That is, deletion of the *ota* cluster occurred in a common ancestor of *A. niger* and *A. welwitschiae*, and resulted in the formation of two alleles: an intact *ota*-cluster allele and a deleted *ota*-cluster allele. Subsequently, as *A. niger* and *A. welwitschiae* diverged from the ancestor, the two alleles were retained by both species. We propose that the second scenario is the most parsimonious, because it requires only one deletion of the cluster and provides a relatively simple explanation for the high level of similarity of An15g07870, An15g07930, and the intergenic region between them in OTA-producing versus nonproducing isolates of *A. niger* and *A. welwitschiae*.

The results of the current study and comparisons with previously published studies provide further insights into the distribution of FB and OTA production among field isolates of the black aspergilli *A. niger* and *A. welwitschiae*. The results also provide evidence for the first time of FB-nonproducing isolates of *A. welwitschiae* with an intact or almost intact *fum* cluster, like the intact *fum* cluster in FB-nonproducing isolates of *A. niger*. The presence of intact *fum* clusters in isolates of *A. niger* and *A. welwitschiae* suggest that such isolates might produce FBs under conditions other than those employed in the current study and other studies (Frisvad et al., 2007, 2011; Palumbo et al., 2013). The relatively high frequency of occurrence of strains that lack the genetic potential to produce either FBs or OTA points to the potential to reduce *Aspergillus*-induced FB/OT contamination of crops by preemptive application of mycotoxin-nonproducing strains of *A. niger* and/or *A. welwitschiae*. That such an approach would be possible is supported by successful control of aflatoxin contamination in multiple crops by preemptive application of aflatoxin-nonproducing isolates of *A. flavus* and *A. parasiticus* (Abbas et al., 2006; Dorner and Horn, 2007). Furthermore, efforts to control *Aspergillus*-induced mycotoxin contamination could be supported by research aimed at understanding the ecological advantage(s) for *Aspergillus* species to exist as mixed populations of mycotoxin-producing and nonproducing individuals.

## Author contributions

AS, AL, AM, RP: Substantial contributions to the conception or design of the work; MM, MH, AG: the acquisition, analysis, or interpretation of data for the work; AS, AL, AM, RP, MM, MH, AG: Drafting the work or revising it critically for important intellectual content; AS, AL, AM, RP, MM, MH, AG: Final approval of the version to be published; AS, AL, AM, RP, MM, MH, AG: Agree for all aspects of the work in ensuring that questions related to the accuracy or integrity of any part of the work are appropriately investigated and resolved.

### Conflict of interest statement

The authors declare that the research was conducted in the absence of any commercial or financial relationships that could be construed as a potential conflict of interest.
