# Supplementary material for: Variation in Fumonisin and Ochratoxin Production Associated with Differences in Biosynthetic Gene Content in Aspergillus niger and A. welwitschiae Isolates from Multiple Crop and Geographic Origins
Source: Front Microbiol. 2016 Sep 9;7:1412. doi: 10.3389/fmicb.2016.01412 (PMC5016791; doi:10.3389/fmicb.2016.01412)

Supplemental Figure S1: Phylogenetic analysis to determine species identity of '*A. phoenicis*' strain ATCC 13157. In a gene tree inferred from concatenated sequences of seven housekeeping genes, strain ATCC 13157 grouped within a clade of *A. niger* strains rather than a clade of *A. welwitschiae* strains. These results indicate that strain ATCC 13157 is a member of the species *A. niger sensu stricto*. The seven housekeeping genes analyzed and proteins they encode were as follows: *dpd1* – DNA Polymerase Delta Subunit; *fasA* – fatty acid synthase alpha subunit; *mcm7* – DNA replication licensing factor; *rpb1* – RNA polymerase largest subunit; *rpb2* – RNA polymerase second largest subunit; *tsr1* – ribosomal biogenesis protein; and *ubt1* – ubiquitin thioesterase. Individual gene sequences retrieved from genome sequences of the 10 strains shown in the figure were aligned using the Muscle function in MEGA5 (Tamura et al. 2011), and then alignments for the seven genes were concatenated. The length of the concatenated alignment was 26,304 nucleotides. The concatenated sequences were subjected to maximum likelihood analysis using the Tamura-Nei substitution model, with gamma distribution and invariant sites, as implemented in MEGA5.

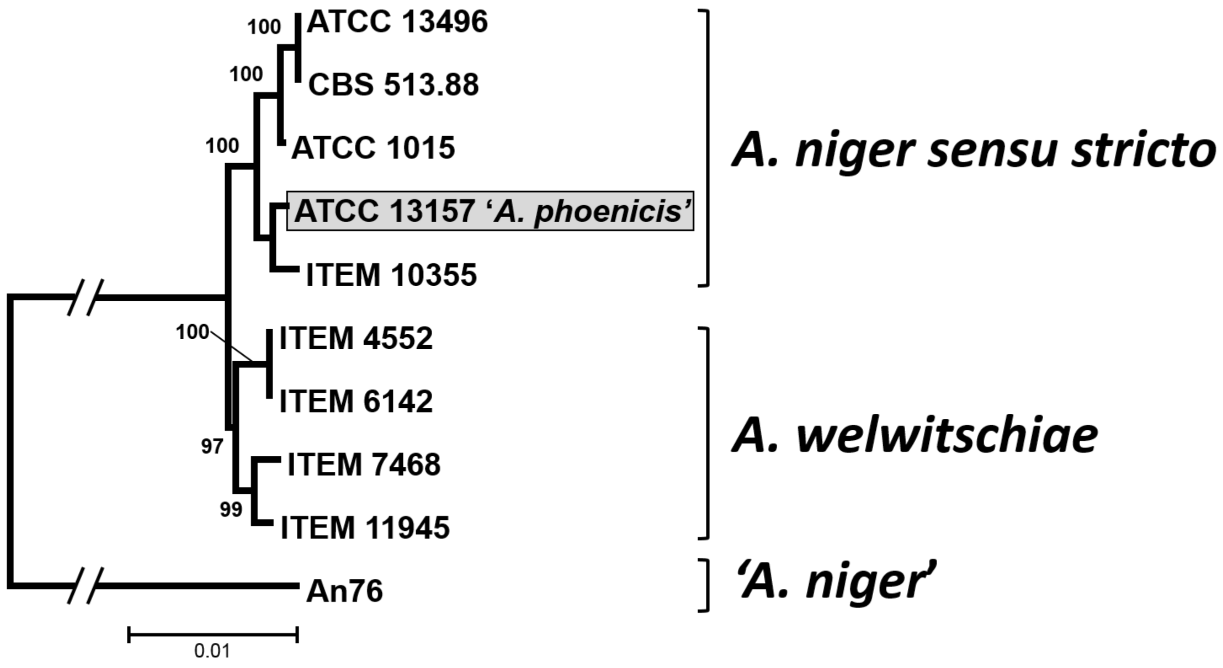

Supplement: Supplementary file 1 [file Image1.PDF]
